# YouTube as an information source in paediatric dentistry education: Reliability and quality analysis

**DOI:** 10.1371/journal.pone.0283300

**Published:** 2023-03-24

**Authors:** İlhan Uzel, Behrang Ghabchi, Ayşe Akalın, Ece Eden

**Affiliations:** 1 Department of Pedodontics, School of Dentistry, Ege University, Izmir, Turkey; 2 Dental Private Practice, Türkiye; The Hong Kong Polytechnic University, HONG KONG

## Abstract

**Introduction:**

In the era of Covid 19 pandemic, the audio-visual contents of YouTube^™^ could be an information source for dental students, practitioners, and patients. The aim of this study was to evaluate the quality, content, and demographics of YouTube^™^ videos about pediatric dentistry for the education of dentistry students.

**Materials and methods:**

A search on YouTube^™^ was performed using the keywords "pediatric dentistry”, “pediatric dental treatments”, “primary teeth treatments" in Turkish. The first 50 videos selected for each keyword were evaluated. Parameters of the videos such as the number of views, the days since the upload, the duration of the video, and the number of likes and dislikes were recorded. Videos are categorized by upload source and content categories as an academic, dentist, physician, patient, reporter, and other, and average points are obtained for the Journal of American Medical Association (JAMA) benchmark. The normality of the data was evaluated with the Shapiro-Wilk test. The data were not distributed normally, compared with the Kruskal Wallis test between source and content groups. The Dunn’s Post Hoc was used to determine to find out which group caused the difference. The Spearman Correlation coefficient was calculated to assess a possible correlation between JAMA, GQS, and VPI scores. All significance levels were set at 0.05.

**Results:**

The duplicates and non-related ones were removed from 150 videos and remaining 119 videos were evaluated. Most of the videos were uploaded by the dentists and other categories, and mainly the videos were uploaded for patient education. JAMA score was 1 out of 4 for 55 videos, 2 for 63 videos, and 3 for only 1 video. When the video source groups were compared, the difference was statistically significant (p = 0.01). The difference between academic and patient groups (p = 0.007); the dentist and patient groups were statistically significant (p = 0.02).

**Conclusion:**

YouTube platform does not contain videos of appropriate quality to support the education of dentistry students in pediatric dentistry in Turkish.

## Introduction

COVID-19 disease (or SARS-CoV-2 virus) is a public health problem that emerged towards the end of 2019 and affected the whole world. This disease, which was declared a “Pandemic” by the World Health Organization on March 11, 2020, has adversely affected many sectors and caused serious problems in both practicing medicine and receiving education in the dental field. With the decision of the government, face-to-face training and exams have been suspended for an indefinite period in our country, as in many countries, due to efforts to minimize social contact to prevent the spread of the new coronavirus. On the other hand, the students had to receive digitalized education and were led to educate themselves—even partially—by obtaining information from various platforms on their own means. Web sites and various social media have rushed to the assistance of students in this regard [1].

Social media is a Web 2.0 technology that was founded in 1979 when Tom Truscott and Jim Ellis of Duke University created a worldwide discussion system that enabled internet users to broadcast public messages [2]. Nowadays, with the development and spread of social media, people have been given the freedom to present their ideas and information in the format they want, and an environment where people are given the opportunity to produce content and access this information easily. In this wide range of topics, many contents related to health are also found. Traditionally, information about medicine and dentistry was available in direct consultation with experts trained in this field. But today, with the use of the internet in every field in developed countries, it has become popular for people to use online resources to access this information [3]. Although the tendency to search for medical information on the internet varies potentially according to age, habit, and location, it has been reported that up to 75% of people use the internet for this purpose [4]. The content provided by social media on health is frequently used not only for informing patients but also for educating students. It has been determined that various educational approaches, especially in social media such as Wikipedia, YouTube ^™^, and Facebook, are frequently used by dentists and medical professionals to get information [5, 6]. Among the changes that have taken place in dental education in recent years, there is also the integration of electronic methods that support multimedia presentations and e-learning strategies in the education of faculty members. A related innovation has been the use of participatory Internet websites, called Web 2.0 content, as they allow academic institutions to provide students with appropriate information with 24/7 accessibility [7]. Since the development and upload of health-related information on the internet are not limited to professionals and it can be done by everyone, it can be assumed that false information, as well as correct information, will also exist [7, 8].

YouTube ^™^, a social media platform where people from all over the world upload, share, and watch videos in a simple and integrated manner was founded in the United States in 2005 by 3 former PayPal employees, Chad Hurley, Steve Chen, and Jawed Karim [9–11].

While YouTube^™^ is the largest and most popular video hosting platform, it is a free video sharing service that is currently the second largest search engine after Google [12, 13].

YouTube ^™^ offers every user the opportunity to upload, watch and share videos, and the site generally includes content such as video clips, televised content, music videos, vlogs (video blogs), short original videos, and educational videos. In YouTube ^™^, both amateurs and professionals can produce content, have their own channel, and communicate with each other through comments [14].

There are many different methods for evaluating the information and content (text, picture, video) uploaded to the internet environment. These methods provide the opportunity to evaluate the content of the website according to different criteria. Among these, The Journal of American Medical Association (JAMA) comparison criteria, Global Quality Score (GQS), and Video Power Index (VPI) are the commonly used evaluation methods.

The Journal of American Medical Association (JAMA) [15] is a non-specific and objective tool consisting of 4 separate criteria, whose benchmarks can be defined in online videos and resources. These criteria provide an assessment of who published the content when it was published, and whether the sources were available. 1 point is earned for each criterion in the videoThe Global Quality Score (GQS) [16] is a specialized, unverified but widely used scoring system in which scores are awarded to determine the quality of online videos [17, 18]. While this scaling system normally uses ordinary people as subjects, in the present study, the Modified Global Quality Score (mGQS), was developed for dentistry by Cesur Aydın et al. [12] was used.

Video Power index (VPI) is a video popularity index based on the number of views and likes of the evaluated videos.

Therefore, the aim of the present study was to evaluate the video contents for dental professionals or students uploaded on YouTube, in terms of the correct information and educational level.

## Materials and methods

The study design has been done as a cross-sectional descriptive study. A search was made by a single researcher (A.A.) under the ’video’ filter using the keywords ’pediatric dentistry, pediatric dental treatments, primary teeth treatments’ on the YouTube ^™^ search engine (https://www.youtube.com/) on 23 November 2020 and it took about one month for analyze and evaluate the videos. The search was carried out by opening a new YouTube ^™^ account that has not been used before. The reason for this is that the YouTube algorithm offers content considering user interactions [19]. While the videos were included in the study, the conditions of being related to the subject were searched. The first 50 videos were evaluated for each keyword.

The title, video source content type, video duration, number of views, days after uploading, and the number of likes and dislikes of each video evaluated were recorded. Using these data, view rate (number of views / days since upload), likes rate [likes / (likes + dislikes) * 100] and video power index (VPI) variables were calculated. The evaluated videos were examined under 6 groups in terms of uploading resources. These are (1) academic (loaders affiliated with research groups and universities); (2) the dentist; (3) physician; (4) the patient; (5) the messenger; (6) other.

Videos were evaluated under 5 titles according to content as: (1) information on undergraduate / postgraduate dental education; (2) patient information; (3) inspection experience; (4) entertainment; (5) advertising. The Journal of American Medical Association (JAMA) comparison criteria [Table 1] were used to evaluate the accuracy and reliability of the videos. A total of 4 points indicates a high source of accuracy and reliability, while 0 points indicate poor source reliability [17]. JAMA comparison criteria are given in Table 1.

**Table 1:** The Journal of American Medical Association (JAMA) comparison criteria.

Criteria	Explanation
Authorship	The credentials and links of the author and contributors should be provided
Attribution	It clearly lists all copyright information, citing references and sources for the content.
Validity	The first date of the posted content and subsequent content updates should be specified.
Explanation	Conflicts of interest, financing, sponsorship, advertising, support, and video ownership should be fully disclosed.

In addition, the quality assessment of the videos was made using the Modified Global Quality Score (mGQS) [Table 2] scaling. This scale scores the quality of the content from 1 to 5. The highest content quality score was determined as 5. More information about the mGQS system is shown in Table 2.

**Table 2:** Modified Global Quality Score (mGQS) scoring system.

Rating	Definition of Quality
1	Poor quality and flow; most of the information is missing, not suitable for use by dentists
2	Generally, low quality and flow; Limited use for dentists as only some information is available
3	Medium quality and low standards of flow; contains some important information but does not provide enough information, useful to the basic level for dentists
4	Good quality and flow; The vast majority of important information on the subject has been presented, useful for dentists
5	Excellent quality and flow; very useful for dentists

Video Power Index is calculated with the formula [like rate * view rate / 100]. The rate of likes is calculated with the formulas [likes * 100 / (likes + dislikes)] and views (number of views/days) [20].

### Statistical analysis

The normality of the data was evaluated with the Shapiro-Wilk test. Since the data were not distributed normally, JAMA, GQS and VPI scores were compared with the Kruskal Wallis test between source groups and content groups. When the difference was statistically significant, the Dunn’s Post Hoc was used to determine to find out which group caused the difference. The Spearman Correlation coefficient was calculated to assess a possible correlation between JAMA, GQS, and VPI scores. The statistical significance level was set at p = 0.05.

## Results

When duplicates were removed from 150 videos that were reached with keywords, a total of 132 videos were evaluated. Among these videos, 13 videos that are not related to the subject were removed. According to the recorded data of the videos that were evaluated, a total of 44282 seconds of video was examined and the average of these videos was calculated as 372 seconds. It has been determined that the videos were liked 14708 times and 3896 disliked by the users in total. Other recorded and calculated properties of the videos are given in Table 3.

**Table 3:** Properties of the examined YouTube videos (n = 119).

	Average	Standard deviation	Minimum	Maximum
Video duration (sec)	372	674	8	5285
Viewed number	46622.21	160953.42	10	1195982
Time from uploading (day)	1222.4	783.45	40	4809
Viewing rate	38.25	133.41	0.01	832.25
Like	124.64	362.2	0	2068
Dislike	33.01	146.83	0	1380
Like ratio (%)	86.95	15.56	0	100

**Fig 1:**
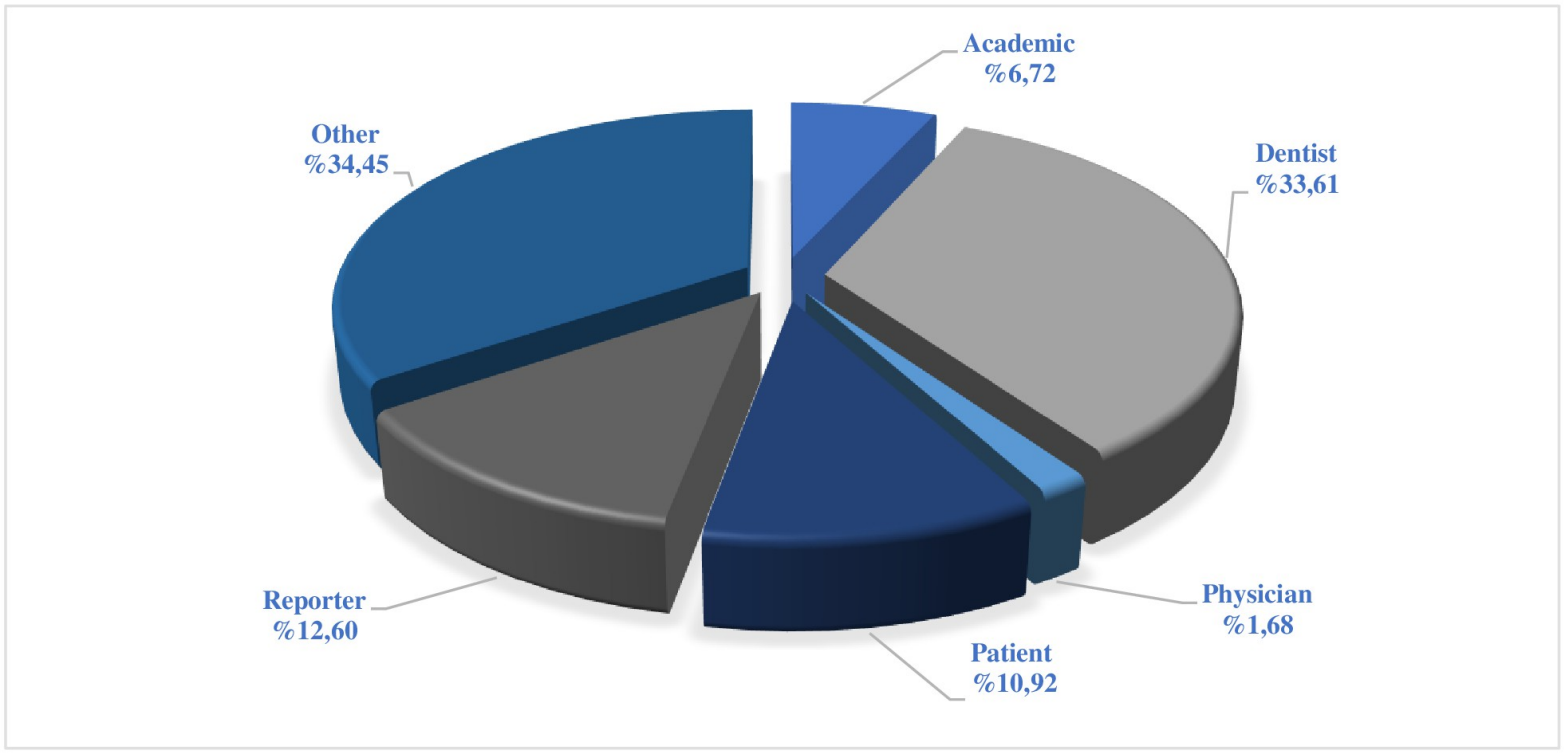
Classification of YouTube videos about pediatric dentistry according to the uploaded source.

According to the video source classification, it was determined that video uploaders had the most accounts in the categories of dentists (33.61% n = 40) and under the category of ‘other’ (34.45% n = 41). It was seen that the least uploaded video source was physicians (1.68% n = 2). The distribution of video sources is given in Fig 1.

**Fig 2:**
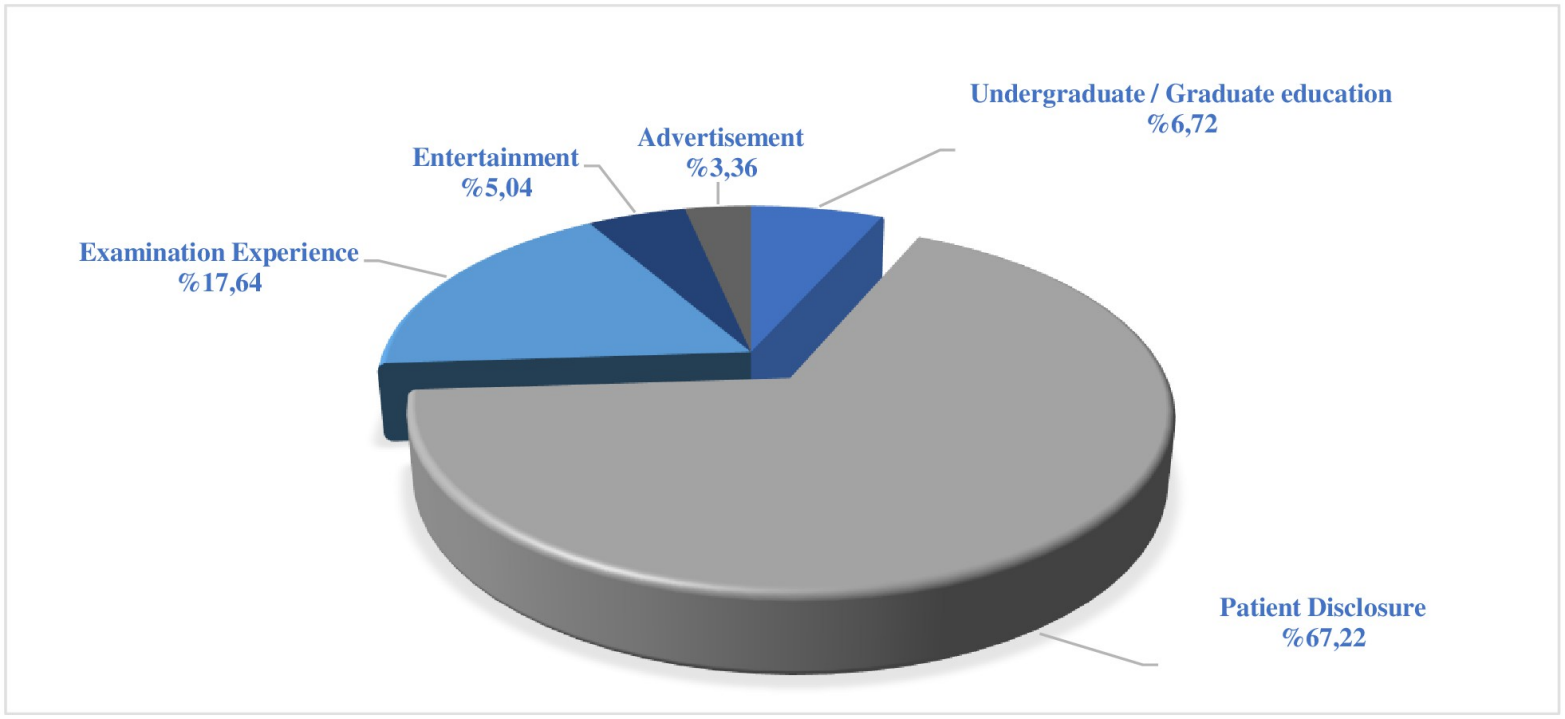
Classification of YouTube videos about pediatric dentistry according to their content.

When the contents of the evaluated videos were examined, it was found that there was a high rate of videos for informing patients (67.22% n = 80) according to the classification made. It was observed that the least uploaded content area was the advertisement (3.36% n = 4) category. The graphic of content classification is given in Fig 2.

### Content

When all sources and contents were evaluated, the mean JAMA score obtained was 1.54 (SD: 0.5), the mean mGQS score was 1.98 (SD: 0.8) and the mean VPI ratio was 34.2 (SD: 108, 2) as calculated. The means and standard deviations (Mean (SD)) of JAMA, mGQS, and VPI values calculated separately for all sources and contents are listed in Table 4.

**Table 4:** Quality and reliability scores of reviewed YouTube videos according to video source and content.

	JAMA	mGQS	VPI
GROUP VARIABLES	Mean (SD)	Mean (SD)	Mean (SD)
**Video Source**			
** Academic**	1.87 (0.3)	3(1.4)	1.64(1.4)
** Dentist**	1.7 (0.5)	2 (0.8)	29.94 (115.7)
** Physician**	2 (0)	2.5 (1.5)	108.4 (0)
** Patient**	1.07 (0.2)	1.07 (0.2)	136.79 (205.05)
** Reporter**	1.53 (0.4)	2.33 (0.6)	15.75 (29.1)
** Other**	1.46 (0.4)	1.90 (0.5)	11.46 (22.9)
**Video content**			
**Undergraduate/ graduate education**	1.87 (0,5)	3.75 (0.8)	22.54 (30.3)
**Patient disclosure**	1.63 (0.4)	2.16 (0.6)	7.55 (17.9)
**Examination experience**	1.19 (0.3)	1.09 (0.2)	105.02 (177.1)
**Entertainment**	1.33 (0.4)	1 (0)	144.06 (249.4)
**Advertisement**	1.25 (0.4)	1 (0)	5.28 (4.3)

When an analysis was made based on the JAMA criteria, it was seen that 55 videos got 1, 63 videos got 2 points and only 1 video got 3 points. None of the videos got a score of 4 indicating full reliability. Although the physicians received the highest score when the uploaded sources of the video were evaluated, these values were quite low to conclude that the videos were of high quality in the reliability criteria. The uploaded videos from academic and dentist sources did not exceed 2 points on average based on these criteria. When the video was examined in terms of content, even though the highest quality was in undergraduate/graduate education, again, no category exceeded 2 points on average. When the video source groups were compared, the difference was statistically significant (p = 0.01). The difference between academic and patient groups (p = 0.007); the dentist and patient groups were statistically significant (p = 0.02). The difference between examination experience and graduate/undergraduate education groups were statistically significant (p = 0.029) in terms of video content.

When the mGQS score was evaluated, it was seen that academic sources uploaded the highest quality videos in terms of video sources. When the videos uploaded by dentists were considered in terms of educational quality, they have fallen behind the journalist and physician resources. Regarding the video content, the highest score in video quality was obtained in the undergraduate/graduate education category as expected. The lowest score was received by the content of entertainment and advertising videos uploaded by patients as a source. There was a statistically significant difference among video source groups in GQS scores (p<0.001). The difference between patient and academic; dentist and reporter groups were statistically significant (p<0.001; p = 0.002; p = 0<0.001, respectively). The difference between graduate/undergraduate content and patient disclosure (p = 0.007), examination experience (p<0.001), entertainment (p<0.001), advertisement (p<0.001) were statistically significant.

When the popularity of videos was examined over the VPI value, it was seen that the videos uploaded by academic education sources had very low popularity, and the most popular videos were uploaded by patients and for entertainment purposes. The difference between academic and reporter groups were statistically significant (p = 0.043) and, dentist and patient groups were significant (p = 0.039). The correlations between JAMA and GQS (r = 0.426); VPI (r = -0.242) scores were low. The correlation between GQS and VPI scores were -0.208. the difference between patient disclose and examinatişon experience was statistically significant (p = 0.002).

## Discussion

Because of the extraordinary situations experienced due to COVID-19, education has shifted towards the home environment and internet-based information acquisition has become almost mandatory. Online videos are among the most preferred of these information resources. There are many reasons why students frequently consult online videos to support their education, such as ease of access, more catchy visual data than written ones, more fun, and no time limit [19]. A considerable part of these online videos is also available on YouTube ^™^, the well-known social media platform [21]. Data in C.H.Basch et al. paper show the potentially inaccurate and negative influence social media can have on population-wide education uptake that should be urgently addressed by agencies of each country’s Public Health Service as well as its global counterparts [22].

However, medical information, including YouTube ^™^, is broadly different from each other in terms of scientific quality, as it is broadcasted directly without any control over the content [23, 24]. As can be seen in this study, most online content uploaders on YouTube ^™^ are people who do not owe academic education and the information they provide is not peer-reviewed and/or validated.

In this study, it has been shown that the sources providing academic education are shared less on the YouTube ^™^ platform than other sources and the average JAMA score of these posts is not sufficient to be considered safe. In terms of content, it has been revealed that although the academic group had, naturally, the highest mGQS score, they offered medium-quality content with an average of 3 points out of 5. When video popularity was examined, this group had a very low rate compared to the others. Although variable parameters such as the number of views and likes of the videos provide us some information about the benefits of the videos, it may be considered normal that the popularity of the academic group was lower than the other groups, since this platform was often a medium with a high demand for entertainment content and open to every user.

It was observed that the videos evaluated within the scope of this study, which was conducted subjectively, were not of high quality and reliable to be used as a source of information in pediatric dentistry education, considering the determined criteria. Although there was not any similar study that evaluated this subject specifically in the literature, the findings of our study were in accordance with earlier studies that reported results of YouTube screening on other dentistry-related issues. Knösel et al. observed that the uploaded video content about dentistry on YouTube did not meet the reliability criteria to a large extent [7]. Similarly, in a study by Burns et al. [25]. 36% of the students reported uncertainty and dissatisfaction in accessing evidence-based information on YouTube.

It can be predicted that online resources will be used frequently during this period, and academic education resources should do their part in this regard. It is necessary to inform students about how to lead them to sources where they can obtain reliable and qualified information, to make more widespread online information sources for the purpose of providing education, and to develop content, with special attention to student education.

## Conclusion

It has been observed that the YouTube ^™^ platform does not contain videos of enough quality and reliability to support the education of dentistry students in pediatric dentistry. The content offered by educators needs to become more widespread and popular. Students should be made aware of which sources to search for and that not all information they obtain from online video sources is completely correct.

## Supporting information

S1 File (DOCX)
