# Supplementary material for: YouTube as an information source in paediatric dentistry education: Reliability and quality analysis
Source: PLoS One. 2023 Mar 24;18(3):e0283300. doi: 10.1371/journal.pone.0283300 (PMC10038246; doi:10.1371/journal.pone.0283300)
Supplement: S1 File — (DOCX) [file pone.0283300.s001.docx]

| **Research Datas** | |
| --- | --- |
| <https://www.youtube.com/watch?v=nAxbKkuoVf4> |  |
| <https://www.youtube.com/watch?v=HV0G1Enntqs> | |
| <https://www.youtube.com/watch?v=RKjJMRjhcXo> | |
| <https://www.youtube.com/watch?v=6inbvBTJWO8> | |
| <https://www.youtube.com/watch?v=aqQ4YzufeLM> | |
| <https://www.youtube.com/watch?v=GcNvA4m7LqA> | |
| <https://www.youtube.com/watch?v=nbOBy-MTWY4> | |
| <https://www.youtube.com/watch?v=jSolQzy-HHw> | |
| <https://www.youtube.com/watch?v=JJ60x-hByfw> | |
| <https://www.youtube.com/watch?v=jQV0T6OLYs0> | |
| <https://www.youtube.com/watch?v=uEyMu6U9ivA> | |
| <https://www.youtube.com/watch?v=xdczAmFSt58> | |
| <https://www.youtube.com/watch?v=qD-ZqYsqdC0> | |
| <https://www.youtube.com/watch?v=SbHuWQaQlQI> | |
| <https://www.youtube.com/watch?v=NmVzMoPd8YI> | |
| <https://www.youtube.com/watch?v=EVXehUmEVik> | |
| <https://www.youtube.com/watch?v=8qmh2l9Jcm0> | |
| <https://www.youtube.com/watch?v=QkaHb84VleI> | |
| <https://www.youtube.com/watch?v=aKow4fbxYQI> | |
| <https://www.youtube.com/watch?v=0A8ri8wDT2M> | |
| <https://www.youtube.com/watch?v=_tcdWOmptj4> | |
| <https://www.youtube.com/watch?v=OAKuSOsVH54> | |
| <https://www.youtube.com/watch?v=7MBRCzxCFjY> | |
| <https://www.youtube.com/watch?v=eFBl2C09cCM> | |
| <https://www.youtube.com/watch?v=HhLsP25c1YY> | |
| <https://www.youtube.com/watch?v=4M5P01_Qg94> | |
| <https://www.youtube.com/watch?v=FI9EwD5iC_8> | |
| <https://www.youtube.com/watch?v=5hez28ewHcU> | |
| <https://www.youtube.com/watch?v=r5LNDQyDslk> | |
| <https://www.youtube.com/watch?v=2Y8uT3l8V8Q> | |
| <https://www.youtube.com/watch?v=mBIP9B6sts0> | |
| <https://www.youtube.com/watch?v=-vdYGBd700E> | |
| <https://www.youtube.com/watch?v=taetX_4Tsb8> | |
| <https://www.youtube.com/watch?v=3DWF5hwz1Vc> | |
| <https://www.youtube.com/watch?v=SctfTsLcygs> | |
| <https://www.youtube.com/watch?v=Hylw-FhYfnQ> | |
| <https://www.youtube.com/watch?v=RJGnVgOV8SU> | |
| <https://www.youtube.com/watch?v=qNXl1Yek64M> | |
| <https://www.youtube.com/watch?v=iN23B1dyCAQ> | |
| <https://www.youtube.com/watch?v=gp7egxn0a7s> | |
| <https://www.youtube.com/watch?v=DgOwE4KNCKc> | |
| <https://www.youtube.com/watch?v=-zfxQteA2Q8> | |
| <https://www.youtube.com/watch?v=gAAullmtTSk> | |
| <https://www.youtube.com/watch?v=TX7ueuxs8Q4> | |
| <https://www.youtube.com/watch?v=uOrj1AoRrO4> | |
| <https://www.youtube.com/watch?v=I0pP29njEcM> | |
| <https://www.youtube.com/watch?v=Z4q9Ka2_cmY> | |
| <https://www.youtube.com/watch?v=W8XM4cPh5Nk> | |
| <https://www.youtube.com/watch?v=tZFg_9D2DhY> | |
| <https://www.youtube.com/watch?v=qxceWNlesOA> | |
| <https://www.youtube.com/watch?v=5cXlZ2BLUjI> | |
| <https://www.youtube.com/watch?v=8RUkRZPWlsE> | |
| <https://www.youtube.com/watch?v=6wDmlaSErzY> | |
| <https://www.youtube.com/watch?v=Q9w41kiVVvA> | |
| <https://www.youtube.com/watch?v=2q068Dj2KLA> | |
| <https://www.youtube.com/watch?v=A5BBcY0Jrno> | |
| <https://www.youtube.com/watch?v=F-gah0RvGrI> | |
| <https://www.youtube.com/watch?v=BMTb76sohgI> | |
| <https://www.youtube.com/watch?v=51BZD47syFs> | |
| <https://www.youtube.com/watch?v=aElNCXaczNE> | |
| <https://www.youtube.com/watch?v=z5fdtE90GPY> | |
| <https://www.youtube.com/watch?v=XjYDWKiI3iM> | |
| <https://www.youtube.com/watch?v=ZjndRvRlfVI> | |
| <https://www.youtube.com/watch?v=fkG0vDDi_IE> | |
| <https://www.youtube.com/watch?v=9QiE35LYiV0> | |
| <https://www.youtube.com/watch?v=4dhYRcWmGIo> | |
| <https://www.youtube.com/watch?v=0aF72Ik_Ubo> | |
| <https://www.youtube.com/watch?v=nR9pKOsdJ4o> | |
| <https://www.youtube.com/watch?v=ewAc4soVnd0> | |
| <https://www.youtube.com/watch?v=cIuVE1cci0A> | |
| <https://www.youtube.com/watch?v=tNiAk4lKxaE> | |
| <https://www.youtube.com/watch?v=0gQymtZvlWs> | |
| <https://www.youtube.com/watch?v=p0keXUPKImg> | |
| <https://www.youtube.com/watch?v=t2TbA08_OF4> | |
| <https://www.youtube.com/watch?v=h-CEmXENTPY> | |
| <https://www.youtube.com/watch?v=0yDZaKLDR8A> | |
| <https://www.youtube.com/watch?v=FyiVVklm4w8> | |
| <https://www.youtube.com/watch?v=qn4We-NDorY> | |
| <https://www.youtube.com/watch?v=o68mOcmj40g> | |
| <https://www.youtube.com/watch?v=dz7pvpqxSnQ> | |
| <https://www.youtube.com/watch?v=xHu2365jdJg> | |
| <https://www.youtube.com/watch?v=fEbnlwrgopo> | |
| <https://www.youtube.com/watch?v=KyVOSV0OrTE> | |
| <https://www.youtube.com/watch?v=KuH5Ilm4Hjg> | |
| <https://www.youtube.com/watch?v=goA7s4Qz2YQ> | |
| <https://www.youtube.com/watch?v=FJBaBwB49_M> | |
| <https://www.youtube.com/watch?v=rT1UlC_KYEE> | |
| <https://www.youtube.com/watch?v=WZlnI9A0BBo> | |
| <https://www.youtube.com/watch?v=zAWsYYJ1S6A> | |
| <https://www.youtube.com/watch?v=ugPOfVQnyvc> | |
| <https://www.youtube.com/watch?v=JlKwTzF4CnY> | |
| <https://www.youtube.com/watch?v=u8jnZLP9CP8> | |
| <https://www.youtube.com/watch?v=9X-RFqNouuE> | |
| <https://www.youtube.com/watch?v=i1uRQgf1bmg> | |
| <https://www.youtube.com/watch?v=_ISzvsSGDqo> | |
| <https://www.youtube.com/watch?v=MLQ9Vyme_fk> | |
| <https://www.youtube.com/watch?v=lIB_Xus7v6g> | |
| <https://www.youtube.com/watch?v=2AxjyYds4V4> | |
| <https://www.youtube.com/watch?v=7HhotgLgigw> | |
| <https://www.youtube.com/watch?v=MosOwHWMzWQ> | |
| <https://www.youtube.com/watch?v=ms9CwEaJWgY> | |
| <https://www.youtube.com/watch?v=p0JhbPKcFLs> | |
| <https://www.youtube.com/watch?v=lXIl_IArflM> | |
| <https://www.youtube.com/watch?v=MDvTkXiLlCM> | |
| <https://www.youtube.com/watch?v=nu20jTwZcMc> | |
| <https://www.youtube.com/watch?v=kwpH_dXJ6vc> | |
| <https://www.youtube.com/watch?v=EWwt4WaWz9U> | |
| <https://www.youtube.com/watch?v=t-GdGExiO-Y> | |
| <https://www.youtube.com/watch?v=OMetfpZJggk> | |
| <https://www.youtube.com/watch?v=rJQTskrJA3A> | |
| <https://www.youtube.com/watch?v=FGdxVaCxKLA> | |
| <https://www.youtube.com/watch?v=To9HHHp_YiU> | |
| <https://www.youtube.com/watch?v=ftqt56L8nYQ> | |
| <https://www.youtube.com/watch?v=uH70Dl3YpHE> | |
| <https://www.youtube.com/watch?v=9CbqtOQF8g0> | |
| <https://www.youtube.com/watch?v=nG4RL9B9KOk> | |
| <https://www.youtube.com/watch?v=xRjLt2LqRu4> | |
| <https://www.youtube.com/watch?v=qCwcPHUhdqA> | |
| <https://www.youtube.com/watch?v=FqGOJOmEqtg> | |
